# Self-reported cancer-related cognitive impairment is associated with perturbed neurotransmission pathways

**DOI:** 10.1007/s00702-024-02824-9

**Published:** 2024-09-26

**Authors:** Kate R. Oppegaard, Yvette P. Conley, Steven Paul, Bruce Cooper, Carolyn S. Harris, Joosun Shin, Lisa Morse, Jon D. Levine, Frances Cartwright, Ritu Roy, Michelle Melisko, Kord M. Kober, Marilyn J. Hammer, Christine Miaskowski

**Affiliations:** 1 Dana-Farber Cancer Institute, University of Massachusetts, Boston, MA USA; 2 School of Nursing, University of Pittsburg, Pittsburgh, PA USA; 3 School of Nursing, University of California, 2 Koret Way – N631Y, San Francisco, CA 94143-0610 USA; 4 The Phyllis F. Cantor Center for Research in Nursing and Patient Care Services, Dana-Farber Cancer Institute, Boston, MA USA; 5 School of Medicine, University of California, San Francisco, CA USA; 6 Mount Sinai Health System, New York, NY USA

**Keywords:** Cancer, Chemotherapy-related cognitive impairment, Cognition, Gene expression, Neurotransmission, Pathway analysis, Patient-reported outcomes

## Abstract

**Background:**

Cancer-related cognitive impairment (CRCI) is reported by 45% of patients with cancer. Significant gaps in knowledge remain regarding the mechanisms that underlie CRCI.

**Objectives:**

Using a data-driven approach, the study purpose was to evaluate for perturbed pathways associated with membership in the High versus the Low CRCI profiles.

**Methods:**

Patients completed the Attentional Function Index six times over two cycles of chemotherapy. Using findings from a previous latent profile analysis, subgroups of patients with high versus low levels of CRCI were evaluated (i.e., High versus Low CRCI profiles). Gene expression was quantified using either ribonucleic (RNA)-sequencing or microarray analyses and pathway impact analyses were performed. Signaling pathways were defined using the Kyoto Encyclopedia of Genes and Genomes database.

**Results:**

A total of 508 patients had data available for analysis. Of the 261 patients in the RNA-sequencing sample, 48.7% were in the High class and 51.3% were in the Low class. Of the 247 patients the microarray sample, 46.6% were in the High class and 53.4% were in the Low class. Pathway impact analyses identified seven perturbed pathways related to neurotransmission (i.e., glutamatergic synapse, GABAergic synapse, dopaminergic synapse, serotonergic synapse, long-term depression, cholinergic synapse, retrograde endocannabinoid signaling).

**Conclusions:**

This study is the first to describe associations between self-reported CRCI in patients receiving chemotherapy for breast, gastrointestinal, gynecological, or lung cancer and seven neurotransmission pathways. These findings provide new insights into potential targets for mechanistically based interventions.

**Supplementary Information:**

The online version contains supplementary material available at 10.1007/s00702-024-02824-9.

## Introduction

Cancer-related cognitive impairment (CRCI) is reported by 45% of patients with cancer (Schmidt et al. 2016). Patients with CRCI report a range of difficulties (e.g., alterations in attention and concentration, decrements in motivation, memory loss) (Mayo et al. 2021). While understood to be complex, significant gaps in knowledge remain regarding the mechanisms that underlie CRCI, which impedes progress in the development of prevention and/or mitigation strategies.

As noted in a review of blood-based biomarkers of CRCI (Oppegaard et al. 2022), while inflammatory mechanisms were the foci for the majority of the biomarker research, evidence suggests that additional mechanisms may contribute to CRCI (e.g., neurodegenerative-processes (Henneghan et al. 2020); alterations in neurotransmission (Lengacher et al. 2015; Barratt et al. 2015). Given this evidence, studies are needed that evaluate multiple mechanisms for CRCI simultaneously. Pathway analysis is one approach that can be used to achieve this objective (García-Campos et al. 2015). Utilizing measures of differential gene expression, pathway analysis evaluates a number of biological factors (e.g., gene-gene interactions) in order to identify perturbed signaling pathways (Mitrea et al. 2013). Subsequently, novel hypotheses can be generated and tested.

In the one study that used pathway analysis to evaluate mechanisms associated with CRCI (Oppegaard et al. 2021), patients receiving chemotherapy were categorized into two groups (i.e., low versus high levels of cognitive function) based on clinically meaningful cutpoints for the Attentional Function Index (AFI) (Cimprich et al. 2011). A total of 12 biological pathways were perturbed (i.e., five related to inflammation, seven related to other processes [data not published]). While informative, the CRCI phenotype was based on a single timepoint (i.e., prior to the second or third cycle of chemotherapy).

For the current pathway analysis, the CRCI phenotype was created using longitudinal data. In our previous study (Atallah et al. 2020), three distinct CRCI profiles were identified (i.e., High (37.2%), Moderate (27.7%), and Low (35.1%) attentional function) and named using the meaningful cutpoints for the AFI (Cimprich et al. 2005). Of the 1329 patients included in this longitudinal study (Atallah et al. 2020), 508 were classified into either the High or Low attentional function profiles and had gene expression data available for analysis. Therefore, in this subset of outpatients receiving chemotherapy, using a data driven approach, the purpose of this study was to evaluate for perturbed pathways associated with membership in the High versus the Low CRCI profiles.

## Methods

### Patients and settings

This study is part of a larger, longitudinal study of the symptom experience of oncology outpatients receiving chemotherapy (Miaskowski et al. 2014). Eligible patients were ≥ 18 years of age; had a diagnosis of breast, gastrointestinal, gynecological, or lung cancer; had received chemotherapy within the preceding four weeks; were scheduled to receive at least two additional cycles of chemotherapy; were able to read, write, and understand English; and gave written informed consent. Patients were recruited from two Comprehensive Cancer Centers, one Veterans Affairs hospital, and four community-based oncology programs.

### Study procedures

The study was approved by the Committee on Human Research at the University of California, San Francisco, and the Institutional Review Board at each of the study sites. Of the 2234 patients approached, 1343 consented to participate (60.1% response rate). The major reason for refusal was being overwhelmed with their cancer treatment. Eligible patients were approached in the infusion unit during their first or second cycle of chemotherapy by a member of the research team to discuss study participation and obtain written informed consent.

Prior to the second or third cycle of chemotherapy, patients completed the AFI (the measure of CRCI in this study) a total of six times over two subsequent cycles of chemotherapy (i.e., prior to chemotherapy administration, approximately one week after chemotherapy administration, approximately two weeks after chemotherapy administration). All of the other measures were done at the enrollment assessment.

At the enrollment assessment, whole blood was collected into PAXgene ribonucleic acid (RNA) stabilization tubes. Total RNA was isolated according to the manufacturer’s standard protocol (Qiagen, USA) using the PaxGene Blood RNA Kit. All of the samples demonstrated a RNA integrity number of ≥ 8 and were retained for gene expression profiling.

Because techniques for quantification of gene expression changed over time, of the 717 patients who provided a blood sample in the parent study, 357 had their samples processed using RNA sequencing (i.e., RNA-seq sample) and 360 had their samples processed using microarray (i.e., microarray sample) technologies (Supplemental Fig. 1). The current study used the gene expression data from patients in the extreme phenotype latent classes that were obtained using the RNA-seq (*n* = 261) and microarray (*n* = 247) technologies.

### Instruments

#### Demographic and clinical characteristics

Patients completed a demographic questionnaire, Karnofsky Performance Status (KPS) scale (Karnofsky et al. 1948), Self-Administered Comorbidity Questionnaire (SCQ) (Sangha et al. 2003), Alcohol Use Disorders Identification Test (AUDIT) (Bohn et al. 1995) and a smoking history questionnaire. The toxicity of each chemotherapy regimen was rated using the MAX2 index (Extermann et al. 2004). Medical records were reviewed for disease and treatment information.

#### CRCI measure

Self-reported CRCI was assessed using the 16-item AFI that assesses an individual’s perceived effectiveness in performing daily activities that are supported by attention, working memory, and executive functions (e.g., setting goals, planning, carrying out tasks) (Cimprich et al. 2011). Higher total mean score (range 0 to 10) indicates greater capacity to direct attention. Clinically meaningful cutpoints for the AFI scores are as follows: <5.0 low function, 5.0 to 7.5 moderate function, and > 7.5 high function (Cimprich et al. 2005). Its Cronbach’s alpha was 0.93.

### Data analysis

#### CRCI phenotyping

In our previous study (Atallah et al. 2020), latent profile analysis (LPA) was used to identify unobserved subgroups of patients (i.e., latent classes) with distinct CRCI profiles over the six assessments using the patients’ scores on the AFI. This LPA identified three subgroups of patients with distinct CRCI profiles (i.e. High (37.2%), Moderate (27.7%) and Low (35.1%) levels of cognitive function). The current study evaluates a subset of patients from the previous LPA (i.e., patients from the High and Low classes) with gene expression data.

#### Imputation of missing demographic and clinical data

Missing data for demographic and clinical characteristics were imputed by the k-nearest-neighbors method, with k = 9. The value of k was selected for zero variance in the bias variance trade-off. For continuous variables, the Euclidean distance was used to find the nearest neighbors. The imputed value was the weighted average of the nearest neighbors, with each weight originally exp(-dist(x, j)), after which the weights were scaled to one. For categorical variables, distance was 0 if the target and the neighbor had the same value and 1 if they did not. The imputed value was the mode of the nearest neighbors.

#### Identification of demographic and clinical characteristics for the logistic regression analyses

The demographic and clinical characteristics of patients in the RNA-seq and microarray samples were analyzed separately. Differences in demographic and clinical characteristics between the patients in the High and Low classes were evaluated using parametric and non-parametric tests. Significance was assessed at a *p*-value of < 0.05.

#### Identification of demographic and clinical characteristics for the gene expression analyses

Separate logistic regression models were done for the RNA-seq and microarray samples. The dependent variable for each of the regression models was membership in the Low attentional function class. In order not to overfit the regression models, the total number of demographic and clinical characteristics selected for evaluation was based on the smaller sample size for the two latent classes. Characteristics included in the final model were selected using a backwards stepwise approach based on the likelihood ratio test. Area under the curve of the receiver operating characteristic curves was used to gauge the overall adequacy of each of the logistic regression models. These analyses were performed using R version 4.0.5.

#### Differential gene expression analyses

The gene expression methods and pathway impact analyses are described in detail elsewhere (Kober et al. 2022). In brief, differential expression was quantified using empirical Bayes models that were implemented using edgeR (Robinson et al. 2010) for the RNA-seq sample and limma (Smyth et al. 2005) for the microarray sample. These analyses were adjusted for select demographic and clinical characteristics that were significantly different between the High and Low classes. In addition, the models included surrogate variables that adjusted for variations due to unmeasured sources. Expression loci were annotated with Entrez gene identifiers. Gene symbols were derived and matched using the HUGO Gene Nomenclature Committee database (Gray et al. 2013). The differential expression results were summarized as the log fold-change and *p*-value for each gene. Only genes that had a common direction of expression across the two samples were retained for subsequent analyses.

#### Pathway impact analyses

The pathway impact analyses included potentially important biological factors (e.g., gene-gene interactions, flow signals in a pathway), as well as the magnitude (i.e., log fold-change) and *p*-values from the differential expression analysis for each sample (Mitrea et al. 2013). The pathway impact analyses included the results of the differential expression analyses for all of the genes (i.e., cutoff free) that had a common direction of differential expression to determine probability of pathway perturbations (pPERT). This analysis was done using Pathway Express version 2.18.0 (Draghici et al. 2007). A total of 225 signaling pathways were found using the Kyoto Encyclopedia of Genes and Genomes (KEGG) database (Aoki-Kinoshita and Kanehisa 2007).

#### Meta-analysis of the pathway impact analyses

For each sample, a separate test was performed for each pathway. Next, Fisher’s combined probability method was used to combine these test results to obtain a single test (global) of the null hypothesis. The significance of the combined transcriptome-wide pathway impact analysis was assessed using a false discovery rate (FDR) of 0.01 under the Benjamini-Hochberg procedure (Dunn 1961).

## Results

### Differences in clinical and demographic characteristics

Of the 261 patients in the RNA-seq sample, 48.7% were in the High class and 51.3% were in the Low class (Table 1). Compared to the High class, the Low class was more likely to be female; less likely to be employed; and had a lower annual income. In addition, the Low class had a lower performance status; a higher number of comorbidities; a higher comorbidity burden; a higher MAX2 score; a lower hematocrit; was less likely to have gastrointestinal cancer and more likely to have lung cancer; and was more likely to self-report diagnoses of lung disease, depression, or back pain.

**Table 1:** Differences in demographic and clinical characteristics at enrollment between patients in the RNA-seq sample with (i.e., low attentional function) and without (high attentional function) CRCI

Characteristic	High attentional function (1) 48.7% (*n* = 127)	Low attentional function (2) 51.3% (*n* = 134)	Statistics
	Mean (SD)	Mean (SD)	
Age (years)	58.0 (11.3)	56.9 (12.4)	t = 0.72, *p* = 0.473
Education (years)	16.2 (3.0)	16.0 (2.9)	t = 0.69, *p* = 0.493
Body mass index (kg/m^2^)	26.3 (4.3)	26.3 (6.3)	t = -0.11, *p* = 0.916
KPS score	82.6 (11.8)	72.4 (11.5)	t = 7.05, *p* < 0.001
Number of comorbidities	2.2 (1.4)	3.1 (1.7)	t = -4.47, *p* < 0.001
SCQ score	4.9 (2.8)	7.2 (3.9)	t = -5.49, < 0.001
AUDIT score	2.8 (2.0)	2.7 (1.8)	t = 0.26, *p* = 0.797
Time since diagnosis (years)	1.6 (2.8)	1.9 (3.5)	U, *p* = 0.954
Time since diagnosis (years, median)	0.5	0.5	
Number of prior cancer treatments	1.6 (1.4)	1.6 (1.5)	t = -0.40, *p* = 0.688
Number of metastatic sites including lymph node involvement	1.3 (1.2)	1.3 (1.3)	t = -0.38, *p* = 0.703
Number of metastatic sites excluding lymph node involvement	0.8 (1.0)	0.9 (1.1)	t = -0.64, *p* = 0.526
MAX2 score	0.16 (0.08)	0.19 (0.08)	t = -2.21, *p* = 0.028
Hemoglobin (g/dL)	11.6 (1.5)	11.3 (1.3)	t = 1.97, *p* = 0.050
Hematocrit (%)	35.0 (4.1)	33.8 (3.8)	t = 2.41, *p* = 0.017
	% (*n*)	% (*n*)	
Gender			
Female Male	67.7 (86) 32.3 (41)	82.8 (111) 17.2 (23)	FE, *p* = 0.006
Ethnicity			
Asian or Pacific Islander Black Hispanic, Mixed, or Other White	15.0 (19) 10.2 (13) 6.3 (8) 68.5 (87)	15.7 (21) 6.0 (8) 14.9 (20) 63.4 (85)	X^2^ = 6.27, *p* = 0.099
Married or partnered (% yes)	61.4 (78)	58.2 (78)	FE, *p* = 0.615
Lives alone (% yes)	24.4 (31)	30.6 (41)	FE, *p* = 0.272
Childcare responsibilities (% yes)	22.0 (28)	17.9 (24)	FE, *p* = 0.440
Care of adult responsibilities (% yes)	6.3 (8)	10.4 (14)	FE, *p* = 0.269
Currently employed (% yes)	46.5 (59)	22.4 (30)	FE, *p* < 0.001
Income			
<$30,000 $30,000 to <$70,000 $70,000 to <$100,000 ≥$100,000	12.6 (16) 19.7 (25) 29.1 (37) 38.6 (49)	27.6 (37) 21.6 (29) 14.2 (19) 36.6 (49)	U, *p* = 0.035 1 > 2
Specific comorbidities (% yes)			
Heart disease High blood pressure Lung disease Diabetes Ulcer or stomach disease Kidney disease Liver disease Anemia or blood disease Depression Osteoarthritis Back pain Rheumatoid arthritis	6.3 (8) 33.1 (42) 3.9 (5) 13.4 (17) 3.1 (4) 0.8 (1) 5.5 (7) 7.9 (10) 7.9 (10) 11.0 (14) 24.4 (31) 3.9 (5)	9.7 (13) 34.3 (46) 17.2 (23) 12.7 (17) 6.0 (8) 0.7 (1) 8.2 (11) 15.7 (21) 36.6 (49) 16.4 (22) 43.3 (58) 5.2 (7)	FE, *p* = 0.367 FE, *p* = 0.896 FE, *p* < 0.001 FE, *p* = 1.000 FE, *p* = 0.378 FE, *p* = 1.000 FE, *p* = 0.468 FE, *p* = 0.057 FE, *p* < 0.001 FE, *p* = 0.215 FE, *p* = 0.002 FE, *p* = 0.770
Exercise on a regular basis (% yes)	67.7 (86)	63.4 (85)	FE, *p* = 0.516
Smoking current or history of (% yes)	35.4 (45)	39.6 (53)	FE, *p* = 0.524
Cancer diagnosis			
Breast Gastrointestinal Gynecological Lung	38.6 (49) 44.9 (57) 11.8 (15) 4.7 (6)	38.8 (52) 27.6 (37) 18.7 (25) 14.9 (20)	X^2^ = 14.21, *p* = 0.003 NS 1 > 2 NS 1 < 2
Type of prior cancer treatment			
No prior treatment Only surgery, CTX, or RT Surgery & CTX, or surgery & RT, or CTX & RT Surgery & CTX & RT	29.1 (37) 37.0 (47) 21.3 (27) 12.6 (16)	25.4 (34) 41.0 (55) 17.2 (23) 16.4 (22)	X^2^ = 1.84, *p* = 0.607
CTX cycle length			
14 day cycle 21 day cycle 28 day cycle	55.1 (70) 34.6 (44) 10.2 (13)	42.5 (57) 47.8 (64) 9.7 (13)	U, *p* = 0.087
Emetogenicity of CTX			
Minimal/low Moderate High	18.1 (23) 66.9 (85) 15.0 (19)	19.4 (26) 58.2 (78) 22.4 (30)	U, *p* = 0.420
Antiemetic regimens			
None Steroid alone or serotonin receptor antagonist alone Serotonin receptor antagonist and steroid NK-1 receptor antagonist and two other antiemetics	5.5 (7) 17.3 (22) 52.0 (66) 25.2 (32)	6.7 (9) 14.2 (19) 49.3 (66) 29.9 (40)	X^2^ = 1.17, *p* = 0.760

Abbreviations: AUDIT = Alcohol Use Disorders Identification Test; CRCI = cancer-related cognitive impairment; CTX = chemotherapy; FE = Fisher’s exact test; kg = kilograms; g/dL = grams per deciliter; KPS = Karnofsky Performance Status; m^2^ = meter squared; NK-1 = neurokinin-1; NS = not significant; RNA-seq = ribonucleic acid sequencing; RT = radiation therapy; SCQ = Self-administered Comorbidity Questionnaire; SD = standard deviation; U = Mann-Whitney U test

Of the 247 patients the microarray sample, 46.6% were in the High class and 53.4% were in the Low class (Table 2). Compared to the High class, the Low class had fewer years of education; was more likely to be female; less likely to be married or partnered; less likely to be employed; less likely to exercise on a regular basis; and had a lower annual income. In addition, the Low class had a lower performance status; a higher number of comorbidities; a higher comorbidity burden; was more likely to self-report a diagnosis of depression or back pain; and less likely to report no prior cancer treatment.

**Table 2:** Differences in demographic and clinical characteristics at enrollment between patients in the microarray sample with (i.e., low attentional function) and without (high attentional function) CRCI

Characteristic	High attentional function (1) 46.6% (*n* = 115)	Low attentional function (2) 53.4% (*n* = 132)	Statistics
	Mean (SD)	Mean (SD)	
Age (years)	58.2 (11.0)	55.7 (12.8)	t = 1.60, *p* = 0.111
Education (years)	17.0 (3.0)	15.9 (2.7)	t = 3.15, *p* = 0.002
Body mass index (kg/m^2^)	26.2 (5.7)	27.5 (6.7)	t = -1.65, *p* = 0.101
KPS score	84.8 (9.8)	76.8 (11.2)	t = 5.91, *p* < 0.001
Number of comorbidities	2.1 (1.1)	2.8 (1.6)	t = -4.21, *p* < 0.001
SCQ score	4.5 (2.2)	6.6 (3.5)	t = -5.58, *p* < 0.001
AUDIT score	2.8 (1.9)	3.3 (2.9)	t = -1.66, *p* = 0.098
Time since diagnosis (years)	2.5 (4.1)	2.4 (3.9)	U, *p* = 0.737
Time since diagnosis (median)	0.5	0.4	
Number of prior cancer treatments	1.8 (1.7)	2.0 (1.7)	t = -1.13, *p* = 0.259
Number of metastatic sites including lymph node involvement	1.4 (1.3)	1.3 (1.2)	t = 1.15, *p* = 0.250
Number of metastatic sites excluding lymph node involvement	1.0 (1.2)	0.8 (1.1)	t = 0.98, *p* = 0.330
MAX2 score	0.16 (0.08)	0.17 (0.08)	t = -0.83, *p* = 0.406
Hemoglobin (g/dL)	12.0 (1.4)	11.8 (1.4)	t = 1.27, *p* = 0.206
Hematocrit (%)	35.7 (4.0)	34.9 (3.9)	t = 1.46, *p* = 0.146
	% (*n*)	% (*n*)	
Gender			
Female Male	72.2 (83) 27.8 (32)	84.1 (111) 15.9 (21)	FE, *p* = 0.029
Ethnicity			
Asian or Pacific Islander Black Hispanic, Mixed, or Other White	9.6 (11) 6.1 (7) 5.2 (6) 79.1 (91)	12.9 (17) 6.8 (9) 12.9 (17) 67.4 (89)	X^2^ = 5.68, *p* = 0.129
Married or partnered (% yes)	69.6 (80)	54.5 (72)	FE, *p* = 0.018
Lives alone (% yes)	20.0 (23)	24.2 (32)	FE, *p* = 0.447
Childcare responsibilities (% yes)	20.9 (24)	29.5 (39)	FE, *p* = 0.144
Care of adult responsibilities (% yes)	6.1 (7)	9.8 (13)	FE, *p* = 0.352
Currently employed (% yes)	46.1 (53)	22.7 (30)	FE, *p* < 0.001
Income			
<$30,000 $30,000 to <$70,000 $70,000 to <$100,000 ≥$100,000	13.9 (16) 17.5 (20) 17.4 (20) 51.3 (59)	34.1 (45) 22.7 (30) 13.6 (18) 29.5 (39)	U, *p* < 0.001 1 > 2
Specific comorbidities (% yes)			
Heart disease High blood pressure Lung disease Diabetes Ulcer or stomach disease Kidney disease Liver disease Anemia or blood disease Depression Osteoarthritis Back pain Rheumatoid arthritis	5.2 (6) 27.8 (32) 11.3 (13) 7.0 (8) 2.6 (3) 0.9 (1) 7.0 (8) 9.6 (11) 12.2 (14) 10.4 (12) 13.9 (16) 1.7 (2)	6.8 (9) 31.1 (41) 12.9 (17) 10.6 (14) 8.3 (11) 1.5 (2) 6.1 (8) 15.9 (21) 34.1 (45) 15.9 (21) 36.4 (48) 3.8 (5)	FE, *p* = 0.791 FE, *p* = 0.675 FE, *p* = 0.846 FE, *p* = 0.374 FE, *p* = 0.058 FE, *p* = 1.000 FE, *p* = 0.801 FE, *p* = 0.183 FE, *p* < 0.001 FE, *p* = 0.261 FE, *p* < 0.001 FE, *p* = 0.454
Exercise on a regular basis (% yes)	77.4 (89)	59.1 (78)	FE, *p* = 0.003
Smoking current or history of (% yes)	33.9 (39)	40.1 (53)	FE, *p* = 0.356
Cancer diagnosis			
Breast Gastrointestinal Gynecological Lung	31.3 (36) 30.4 (35) 23.5 (27) 14.8 (17)	40.2 (53) 26.5 (35) 23.5 (31) 9.8 (13)	X^2^ = 2.90, *p* = 0.407
Type of prior cancer treatment			
No prior treatment Only surgery, CTX, or RT Surgery & CTX, or surgery & RT, or CTX & RT Surgery & CTX & RT	24.3 (28) 35.7 (41) 25.2 (29) 14.8 (17)	12.1 (16) 45.5 (60) 20.5 (27) 22.0 (29)	X^2^ = 8.92, *p* = 0.030 1 > 2 NS NS NS
CTX cycle length			
14 day cycle 21 day cycle 28 day cycle	39.1 (45) 52.2 (60) 8.7 (10)	34.1 (45) 59.1 (78) 6.8 (9)	U, *p* = 0.595
Emetogenicity of CTX			
Minimal/low Moderate High	23.5 (27) 62.6 (72) 13.9 (16)	27.3 (36) 59.1 (78) 13.6 (18)	U, *p* = 0.588
Antiemetic regimens			
None Steroid alone or serotonin receptor antagonist alone Serotonin receptor antagonist and steroid NK-1 receptor antagonist and two other antiemetics	13.9 (16) 22.6 (26) 47.8 (55) 15.7 (18)	9.1 (12) 22.7 (30) 43.2 (57) 25.0 (33)	X^2^ = 4.15, *p* = 0.245

Abbreviations: AUDIT = Alcohol Use Disorders Identification Test; CRCI = cancer-related cognitive impairment; CTX = chemotherapy; FE = Fisher’s exact test; g/dL = grams per deciliter; kg = kilograms; KPS = Karnofsky Performance Status; m^2^ = meter squared; NK-1 = neurokinin-1; NS = not significant; RT = radiation therapy; SCQ = Self-administered Comorbidity Questionnaire; SD = standard deviation; U = Mann-Whitney U test

### Demographic and clinical characteristics included in the gene expression analyses

In the logistic regression analysis for the RNA-seq sample (Table 3), ten characteristics were included in the initial model and six were retained in the final model and were used as covariates in the gene expression analysis (i.e., male gender, being employed, MAX2 score, KPS score, SCQ score, self-reported diagnosis of depression). In the logistic regression analysis for the microarray sample, ten characteristics were included in the initial model and seven were retained in the final model and used as covariates in the gene expression analysis (i.e., years of education, being employed, exercise on regular basis, KPS score, self-reported diagnosis of depression, self-reported diagnosis of back pain, type of prior cancer treatment).

**Table 3:** Multiple logistic regression analyses predicting membership in the low attentional function class

RNA-sequencing sample (*n* = 261)			
Predictors	Odds Ratio	95% CI	*p*-value
Male gender	0.43	0.21, 0.87	0.021
Employed	0.36	0.19, 0.67	0.002
MAX2 score	30.04	0.67, 1506.43	0.083
Karnofsky Performance Status score	0.95	0.92, 0.97	< 0.001
Self-administered comorbidity score	1.14	1.03, 1.26	0.015
Self-reported diagnosis of depression	3.58	1.55, 8.81	0.004
Overall model fit: AUC of the ROC = 0.822			
Microarray sample (*n* = 247)			
Predictors	Odds Ratio	95% CI	*p*-value
Years of education	0.90	0.81, 1.00	0.064
Employed	0.58	0.30, 1.09	0.092
Exercise on a regular basis	0.51	0.26, 0.98	0.043
Karnofsky Performance Status score	0.95	0.92, 0.98	0.001
Self-reported diagnosis of depression	2.38	1.13, 5.19	0.022
Self-reported diagnosis of back pain	2.80	1.38, 5.92	0.004
Type of prior cancer treatment			
No prior treatment Only surgery, CTX, or RT Surgery & CTX, or surgery & RT, or CTX & RT Surgery & CTX & RT	1.00 2.70 1.48 3.15	1.19, 6.35 0.59, 3.77 1.18, 8.75	0.043
Overall model fit: AUC of the ROC = 0.793			

Abbreviations: AUC = area under curve; CI = confidence interval; CTX = chemotherapy; ROC = receiver operating characteristic; RT = radiotherapy

### Differential gene expression analyses

For the RNA-seq sample, median library threshold size was 9,402,364 reads. Following the application of quality control filters, 13,936 genes were included in the final analysis. The common dispersion was estimated as 0.191, yielding a biological coefficient of variation of 0.438.

For the microarray sample, all of the samples demonstrated good hybridization performance for biotin, background negative, and positive control assays on the arrays. Following quality control filters, 43,933 loci were included in the final analysis.

### Pathway impact analyses

For the RNA-seq sample, two surrogate variables were identified and included in the final differential expression model. For the microarray sample, zero surrogate variables were identified. For both samples, a total of 4,529 genes were included in the pathway impact analyses. Across the two samples, 79 KEGG signaling pathways were significantly perturbed at an FDR of < 0.01 (Supplemental Table 1). For this paper, the seven perturbed pathways related to neurotransmission are discussed (Table 4).

**Table 4:** Significantly perturbed neurotransmission-related pathways

Pathway ID	Pathway name	Combined analysis statistics
hsa04724	Glutamatergic synapse	X^2^ = 23.18, *p* = 0.001
hsa04727	GABAergic synapse	X^2^ = 17.64, *p* = 0.006
hsa04728	Dopaminergic synapse	X^2^ = 21.47, *p* = 0.002
hsa04726	Serotonergic synapse	X^2^ = 18.00, *p* = 0.006
hsa04730	Long-term depression	X^2^ = 16.90, *p* = 0.007
hsa04725	Cholinergic synapse	X^2^ = 16.53, *p* = 0.008
hsa04723	Retrograde endocannabinoid signaling	X^2^ = 30.41, *p* < 0.001

Note: *p* = global perturbation *p*-value adjusted using the Benjamini-Hochberg procedure

Abbreviations: hsa = homo sapiens; ID = identifier

## Discussion

This study is the first to describe associations between self-reported CRCI in patients receiving chemotherapy for breast, gastrointestinal, gynecological, or lung cancer and seven neurotransmission pathways (i.e., glutamatergic synapse, GABAergic synapse, dopaminergic synapse, serotonergic synapse, long-term depression, cholinergic synapse, retrograde endocannabinoid signaling). Of note, evidence suggests that chemotherapy induces changes in neurotransmission within 24 h of administration (Thomas et al. 2017). Given this evidence and the fact that patients in this study had received at least one cycle of chemotherapy supports the hypothesis that alterations in neurotransmission may contribute to CRCI. Differences between the classes in demographic and clinical characteristics were discussed in the previous publication (Atallah et al. 2020). This discussion focuses on associations between chemotherapy-related cognitive changes and the neurotransmitters associated with these pathways.

### Glutamatergic synapse

Glutamine is the amino acid precursor of glutamate and γ-aminobutyric acid (GABA). Given that 90% of neurons have glutamate receptors, it is the most common neurotransmitter within the central nervous system (Baek et al. 2024). In terms of its association with CRCI, in a study of mice treated with a single dose of doxorubicin compared to controls (Thomas et al. 2017), glutamate uptake was 45% slower in the frontal cortex and took 48% longer to clear the dentate gyrus 24 h post administration. The authors concluded that receipt of doxorubicin alters glutamate neurotransmission in nuclei associated with cognitive function within 24 h of chemotherapy administration. In another study that evaluated the acute effects of doxorubicin (Alhowail et al. 2019), a dose-dependent association was found with impairments in long-term potentiation of hippocampal neurons. The authors noted that long-term potentiation in a complex process that is dependent on hippocampal glutamatergic neurotransmission.

### GABAergic synapse

GABA is a key modulator of brain function through its regulation of the inhibitory-excitatory balance between ionotropic and metabotropic GABA receptors (McArdle et al. 2023). In terms of CRCI, in a study of mice that used a network-based analysis to identify amino acid signatures in brain regions affected by systemic doxorubicin treatment (Liu et al. 2023), GABA was a potential signature in brain regions associated with cognitive function (e.g., hippocampus, neocortex, prefrontal cortex). In addition, compared to controls, cognitive impairment was observed in the treated mice. Of note, this study identified that doxorubicin treatment altered the levels of other amino acids (i.e., phenylalanine, tyrosine, methionine) in the brain through liver and kidney damage. These authors suggested that the cognitive impairment associated with doxorubicin may be partially due to damage to other organs.

### Dopaminergic synapse

Dopamine is a neurotransmitter that is involved in multiple pathways in the brain (e.g., movement, reward) (Juárez Olguín et al. 2016). In a study of rats given 5-fluorouracil (Jarmolowicz et al. 2019), the treatment impaired attentional shifting and caused a decrease in dopamine release. These results suggest that alterations in dopamine release may contribute to CRCI. In a study that evaluated cortical and hippocampal neurochemical changes induced by doxorubicin in rats (Khadrawy et al. 2021), cortical dopamine levels increased significantly after the injection of chemotherapy. The authors concluded that increased dopamine levels may impair cogntive function through multiple pathways (e.g., induction of oxidative stress and/or inflammation).

### Serotonergic synapse

The neurotransmitter serotonin regulates a variety of biological functions (e.g., cognition, mood) (Kalinichenko et al. 2024). In a study of rats treated with carboplatin for four weeks (Kaplan et al. 2016), serotonin release was impaired and decrements in spatial learning discrimination were observed. In addition, dopamine release and uptake were impaired. However, overall dopamine content and the reserve pool of dopamine did not change significantly. Taken together, these results suggest that chemotherapy selectively impairs dopamine release and uptake processes and serotonin release that contribute to impairments in learning.

### Additional pathways

While in this study, both the long-term depression and cholinergic pathways were perturbed, no studies were identified that reported on associations between CRCI and either of these pathways. However, in a study that aimed to comprehensively understand the context and distribution of pathways that contribute to Alzheimer’s disease (Morgan et al. 2022), text-mining was used to generate a systematic assessment of the breadth and diversity of biological pathways within a corpus of 206,324 dementia-related publications. Across research spanning 30 years, the long-term depression and cholinergic synapse pathways were consistently associated with Alzheimer’s disease, which suggests that these two pathways are important regulators of brain function.

The endocannabinoid system regulates retrograde signaling in the central nervous system through the modulation of neurotransmitters and synaptic plasticity (Kibret et al. 2023). No studies were identified that reported on associations between CRCI and the retrograde endocannabinoid signaling pathway. However, in a study that evaluated for associations between differential serum metabolites and their corresponding pathways in patients with Alzheimer’s disease (Yu et al. 2023), this pathway was enhanced and the GABAergic synapse pathway was downregulated.

### Associations between CRCI and neurotransmission-related genes

While an evaluation of individual genes within these perturbed pathways is beyond the scope of the current study, it is worth noting that evidence from other studies suggests that a variety of neurotransmitter-related genes may contribute to CRCI. For example, catechol-O-methyltransferase (COMT) is an enzyme responsible for regulation of catecholamine neurotransmitters (e.g., dopamine) (Qayyum et al. 2015). In a study of patients with breast cancer (Cheng et al. 2016), compared to patients with the GG genotype for *COMT* rs165599, patients with the GA and AA genotypes had a lower odds of developing self-reported cognitive decline. However, in two studies of patients with cancer (Lengacher et al. 2015; Barratt et al. 2015), no associations were found between subjective or objective measures of CRCI and *COMT* rs4860.

The solute carrier 6 family group of genes includes transporters for a variety of neurotransmitters (e.g., GABA, serotonin, dopamine) (Bröer and Gether 2012). In a study of breast cancer survivors (Lengacher et al. 2015), individuals who were homozygous for the common G allele (GG) in *solute carrier family 6 member 4* (*SLC6A)* rs16965628 had greater improvements in three cognitive outcomes (i.e., memory, organization, global cognition) compared to individuals who were heterozygous or homozygous for the rare C allele (GC or CC).

Finally, in a study of patients with breast or prostate cancer (Harris et al. 2023), associations were found between the severity of three distinct symptom clusters (i.e., sickness-behavior, mood-cognitive, treatment-related) and polymorphisms for 16 genes involved in neurotransmission. While CRCI was not evaluated as an individual symptom, associations were reported between the mood-cognitive cluster and genes involved in catecholaminergic (i.e., *adrenoreceptor alpha 1D*), GABAergic (*SLC6A1*), and serotonergic (*SLC6A2*, *SLC6A3*, *5-hydroxytryptamine receptor* (*HTR*) *2 A*, and *HTR3A*) neurotransmission. Overall, these studies provide additional support for our data-driven hypothesis that alterations in neurotransmission contribute to CRCI.

### Limitations

Because patients in this study had a diagnosis of breast, gastrointestinal, gynecological, or lung cancer, our findings may not generalize to patients with other types of cancer. In addition, because an evaluation cohort was not used, these findings warrant confirmation in an independent sample. Because CRCI was assessed using a self-report measure, an evaluation using objective measures may yield different CRCI profiles. Although our CRCI phenotype was created based on longitudinal assessments, blood was collected only at the enrollment assessment. Longitudinal studies are needed that collect both symptom and molecular data to determine if pathway perturbations change over time and to determine the direction for these associations. Equally important, future studies should evaluate for pathway perturbations in other tissues (e.g., cerebral spinal fluid) and/or for associations with additional biomarkers (e.g., genetic, epigenetic).

## Conclusions and implications for future research

This data-driven study provides new information on associations between self-reported CRCI and pathways involved in neurotransmission. Each of these perturbed pathways represents a complex biological process. Therefore, additional research is needed to determine how one or more of these pathways act collectively to contribute to CRCI. In addition, specific molecular targets, that are common across these pathways, should be evaluated in future studies (e.g., candidate gene analyses). In addition, future studies can evaluate for associations between CRCI and pathway perturbations within and across cancer types and chemotherapy regimens. While these findings warrant confirmation, they provide novel information on associations between self-reported CRCI and neurotransmission-related mechanisms that can be used to inform future research and provide insights into potential targets that can be used to develop interventions.

## Electronic supplementary material

Below is the link to the electronic supplementary material.

Supplementary Material 1

Supplementary Material 2

## Data Availability

Data are available from the corresponding author after the completion of a material transfer agreement with the University of California, San Francisco.
