# Supplementary material for: Self-reported cancer-related cognitive impairment is associated with perturbed neurotransmission pathways
Source: J Neural Transm (Vienna). 2024 Sep 26;132(2):275–86. doi: 10.1007/s00702-024-02824-9 (PMC11785672; doi:10.1007/s00702-024-02824-9)
Supplement: Supplementary file 1 — Supplementary Material 1 [file 702_2024_2824_MOESM1_ESM.docx]

1329 patients with cancer-related cognitive impairment phenotypic data in the parent study by latent class:

High attentional function (n=495); Moderate attentional function (n=368); Low attentional function (n=466)

Excluded patients in the Moderate class

n = 107

Supplementary Figure 1: Flow diagram of the number of patients available for the gene expression analyses that evaluated for perturbations between the High attentional function and Low attentional function latent classes.

Abbreviations: GE = gene expression; RNA-seq = ribonucleic acid sequencing

Excluded patients in the Moderate class

n = 89

Evaluable patients

High class = 115

Low class = 132

Evaluable patients

High class = 127

Low class = 134

GE analysis

Phenotypic analysis

Evaluable patients

n = 247

Evaluable patients

n = 261

Excluded after imputation n = 0

Microarray sample

n = 247

Excluded for poor quantification n = 6

Microarray sample

n = 253

717 provided a blood sample

Microarray sample

n = 360

Quality control for phenotypic data

Patients with phenotypic data

Patients with the extreme phenotypes

Quality control for GE methodology

Excluded after imputation n = 0

RNA-seq sample

n = 261

Excluded for poor quantification n = 7

RNA-seq sample

n = 268

GE methodology

RNA-seq sample

n = 357
