# Supplementary material for: Self-reported cancer-related cognitive impairment is associated with perturbed neurotransmission pathways
Source: J Neural Transm (Vienna). 2024 Sep 26;132(2):275–86. doi: 10.1007/s00702-024-02824-9 (PMC11785672; doi:10.1007/s00702-024-02824-9)
Supplement: Supplementary file 2 — Supplementary Material 2 [file 702_2024_2824_MOESM2_ESM.docx]

Supplemental Table 1. Pathway Impact Analysis Results for the Low CRCI Versus High CRCI Classes

| Pathway ID | KEGG Pathway Name | pPert  RNA-seq | pPert  micora | Global X^2^ | Global FDR |
| --- | --- | --- | --- | --- | --- |
| hsa04260 | Cardiac muscle contraction | <0.001 | <0.001 | 30.41 | <0.001 |
| hsa04723 | Retrograde endocannabinoid signaling | <0.001 | <0.001 | 30.41 | <0.001 |
| hsa05020 | Prion disease | <0.001 | <0.001 | 30.41 | <0.001 |
| hsa05022 | Pathways of neurodegeneration - multiple diseases | <0.001 | <0.001 | 30.41 | <0.001 |
| hsa05132 | Salmonella infection | <0.001 | 0.001 | 28.21 | <0.001 |
| hsa05010 | Alzheimer disease | <0.001 | 0.002 | 27.63 | <0.001 |
| hsa05016 | Huntington disease | 0.002 | <0.001 | 27.63 | <0.001 |
| hsa05014 | Amyotrophic lateral sclerosis | 0.002 | <0.001 | 27.19 | 0.001 |
| hsa04145 | Phagosome | 0.003 | <0.001 | 26.82 | 0.001 |
| hsa04261 | Adrenergic signaling in cardiomyocytes | <0.001 | 0.003 | 26.51 | 0.001 |
| hsa05164 | Influenza A | <0.001 | 0.003 | 26.51 | 0.001 |
| hsa05202 | Transcriptional misregulation in cancer | 0.001 | 0.003 | 25.44 | 0.001 |
| hsa04144 | Endocytosis | 0.001 | 0.002 | 24.99 | 0.001 |
| hsa04270 | Vascular smooth muscle contraction | <0.001 | 0.010 | 24.32 | 0.001 |
| hsa04360 | Axon guidance | <0.001 | 0.011 | 24.13 | 0.001 |
| hsa05012 | Parkinson disease | 0.011 | <0.001 | 24.13 | 0.001 |
| hsa04024 | cAMP signaling pathway | <0.001 | 0.012 | 23.97 | 0.001 |
| hsa04010 | MAPK signaling pathway | <0.001 | 0.013 | 23.89 | 0.001 |
| hsa04151 | PI3K-Akt signaling pathway | 0.002 | 0.004 | 23.47 | 0.001 |
| hsa04810 | Regulation of actin cytoskeleton | <0.001 | 0.016 | 23.41 | 0.001 |
| hsa04613 | Neutrophil extracellular trap formation | 0.018 | <0.001 | 23.24 | 0.001 |
| hsa05165 | Human papillomavirus infection | 0.004 | 0.002 | 23.24 | 0.001 |
| hsa04724 | Glutamatergic synapse | <0.001 | 0.018 | 23.18 | 0.001 |
| hsa05160 | Hepatitis C | <0.001 | 0.027 | 22.39 | 0.002 |
| hsa05131 | Shigellosis | 0.006 | 0.002 | 22.22 | 0.002 |
| hsa04072 | Phospholipase D signaling pathway | 0.001 | 0.010 | 22.12 | 0.002 |
| hsa04022 | cGMP-PKG signaling pathway | <0.001 | 0.033 | 22.03 | 0.002 |
| hsa04066 | HIF-1 signaling pathway | 0.017 | 0.001 | 21.97 | 0.002 |
| hsa05200 | Pathways in cancer | 0.002 | 0.007 | 21.91 | 0.002 |
| hsa04060 | Cytokine-cytokine receptor interaction | 0.020 | 0.001 | 21.64 | 0.002 |
| hsa04728 | Dopaminergic synapse | 0.001 | 0.014 | 21.47 | 0.002 |
| hsa05171 | Coronavirus disease - COVID-19 | 0.044 | <0.001 | 21.45 | 0.002 |
| hsa04621 | NOD-like receptor signaling pathway | 0.006 | 0.004 | 21.28 | 0.002 |
| hsa04930 | Type II diabetes mellitus | <0.001 | 0.048 | 21.28 | 0.002 |
| hsa05152 | Tuberculosis | 0.013 | 0.002 | 21.12 | 0.002 |
| hsa04713 | Circadian entrainment | <0.001 | 0.052 | 21.12 | 0.002 |
| hsa04218 | Cellular senescence | 0.002 | 0.016 | 20.26 | 0.003 |
| hsa05150 | Staphylococcus aureus infection | 0.012 | 0.003 | 20.08 | 0.003 |
| hsa04929 | GnRH secretion | <0.001 | 0.101 | 19.79 | 0.003 |
| hsa05310 | Asthma | 0.002 | 0.027 | 19.62 | 0.003 |
| hsa05323 | Rheumatoid arthritis | 0.012 | 0.004 | 19.57 | 0.003 |
| hsa04925 | Aldosterone synthesis and secretion | 0.001 | 0.062 | 19.36 | 0.004 |
| hsa04932 | Non-alcoholic fatty liver disease | 0.016 | 0.004 | 19.25 | 0.004 |
| hsa04928 | Parathyroid hormone synthesis, secretion and action | 0.007 | 0.009 | 19.10 | 0.004 |
| hsa05207 | Chemical carcinogenesis - receptor activation | <0.001 | 0.153 | 18.96 | 0.004 |
| hsa05144 | Malaria | 0.003 | 0.028 | 18.77 | 0.004 |
| hsa04217 | Necroptosis | 0.187 | <0.001 | 18.55 | 0.005 |
| hsa05415 | Diabetic cardiomyopathy | 0.217 | <0.001 | 18.26 | 0.005 |
| hsa04726 | Serotonergic synapse | <0.001 | 0.246 | 18.00 | 0.006 |
| hsa04620 | Toll-like receptor signaling pathway | 0.044 | 0.003 | 17.87 | 0.006 |
| hsa04921 | Oxytocin signaling pathway | 0.001 | 0.134 | 17.84 | 0.006 |
| hsa04970 | Salivary secretion | 0.021 | 0.006 | 17.75 | 0.006 |
| hsa05145 | Toxoplasmosis | 0.047 | 0.003 | 17.74 | 0.006 |
| hsa05133 | Pertussis | 0.071 | 0.002 | 17.71 | 0.006 |
| hsa04727 | GABAergic synapse | <0.001 | 0.296 | 17.64 | 0.006 |
| hsa05017 | Spinocerebellar ataxia | 0.024 | 0.006 | 17.53 | 0.006 |
| hsa05417 | Lipid and atherosclerosis | 0.002 | 0.079 | 17.5 | 0.006 |
| hsa05206 | MicroRNAs in cancer | 0.004 | 0.04 | 17.48 | 0.006 |
| hsa04924 | Renin secretion | <0.001 | 0.378 | 17.15 | 0.007 |
| hsa04973 | Carbohydrate digestion and absorption | <0.001 | 0.389 | 17.09 | 0.007 |
| hsa04020 | Calcium signaling pathway | <0.001 | 0.411 | 16.98 | 0.007 |
| hsa05414 | Dilated cardiomyopathy | <0.001 | 0.417 | 16.95 | 0.007 |
| hsa05134 | Legionellosis | 0.212 | 0.001 | 16.92 | 0.007 |
| hsa04730 | Long-term depression | 0.001 | 0.143 | 16.9 | 0.007 |
| hsa04668 | TNF signaling pathway | 0.006 | 0.034 | 16.84 | 0.007 |
| hsa05031 | Amphetamine addiction | <0.001 | 0.49 | 16.63 | 0.008 |
| hsa04146 | Peroxisome | 0.031 | 0.008 | 16.61 | 0.008 |
| hsa04725 | Cholinergic synapse | <0.001 | 0.514 | 16.53 | 0.008 |
| hsa05140 | Leishmaniasis | 0.549 | <0.001 | 16.4 | 0.008 |
| hsa05322 | Systemic lupus erythematosus | 0.093 | 0.003 | 16.36 | 0.008 |
| hsa04912 | GnRH signaling pathway | <0.001 | 0.616 | 16.17 | 0.009 |
| hsa04014 | Ras signaling pathway | 0.006 | 0.047 | 16.17 | 0.009 |
| hsa04927 | Cortisol synthesis and secretion | <0.001 | 0.669 | 16.01 | 0.009 |
| hsa04137 | Mitophagy - animal | 0.009 | 0.039 | 15.91 | 0.009 |
| hsa05412 | Arrhythmogenic right ventricular cardiomyopathy | <0.001 | 0.711 | 15.89 | 0.009 |
| hsa04934 | Cushing syndrome | 0.001 | 0.239 | 15.86 | 0.009 |
| hsa04350 | TGF-beta signaling pathway | 0.007 | 0.048 | 15.86 | 0.009 |
| hsa05410 | Hypertrophic cardiomyopathy | <0.001 | 0.777 | 15.71 | 0.010 |
| hsa05167 | Kaposi sarcoma-associated herpesvirus infection | 0.007 | 0.057 | 15.64 | 0.010 |

Note: Global FDR adjusted using the Benjamini-Hochberg procedure

Abbreviations: CRCI = cancer-related cognitive impairment; FDR = false discovery rate; has = homo sapiens; ID = identifier; KEGG = Kyoto Encyclopedia of Genes and Genomes; microa = microarray sample; pPert = probability of pathway perturbations; RNA-seq = ribonucleic acid sequencing sample
